# Method of Inducing Local Anæsthesia by Cocaine

**Published:** 1893-10

**Authors:** D. Caracatsanis

**Affiliations:** Athens, Greece


### METHOD OF INDUCING LOCAL ANÆSTHESIA BY COCAINE.[1]

BY D. CARACATSANIS, M.D., ATHENS, GREECE.

You are all acquainted with the danger of cocaine-injections for the painless extraction of teeth. Its narcotic effects are surprising, but unhappily they are frequently followed by untoward consequences; even loss of life has occasionally resulted. At the Dental Congress in Paris animated discussions arose on this subject. Since then I undertook some experiments to determine whether some procedure might not be substituted for injection, some milder method for the induction of local anæthesia by means of cocaine.

I proceeded on the basis that if its effects could be completely localized, cocaine would answer perfectly. Such a method I have found; I have applied it practically for several years. The patients have nothing to complain of, not even the slightest indisposition. Accordingly it is with the greatest confidence in my method that I lay it before you. The operation is simple, within the power of every one; its only imperfection is that its application demands considerable time; sometimes as much as three-quarters of an hour is required for complete anæsthesia. The procedure is as follows: I begin painting the gum, next the tooth to be extracted, with a steel instrument wrapped in cotton dipped in a solution of phenic acid, 2 to 1000, which I have heated. This is followed by the application of the salt of cocaine by means of a pledget of cotton impregnated therewith. As soon as the gum shows signs of insensibility, I commence to separate it slowly from the tooth by means of a bistoury. I insert into the space thus effected pledgets of cotton impregnated with cocaine as before. As the anæsthesia advances I enlarge the opening to a depth of about one centimetre, on the buccal as well as on the lingual surface. I direct the patient to abstain from swallowing the saliva, to avoid all absorption of cocaine. I take good care not to forget the cotton pledgets placed between the gum and the tooth.

After assuring myself by strongly making pressure on the parts with a steel instrument, I have my assistant to spray the parts with a mixture composed as follows:

---

[1] Read before the World's Columbian Dental Congress, Chicago, August, 1893.

Chloroform, 25 grammes;
Sulphuric ether, 40 grammes;
Menthol, 3 grammes;
Cocaine, 1 gramme;
Essence of mint, 1 gramme.

I extract while the parts are being sprayed. The resulting anæthesia is absolutely complete; the only condition in which I have failed to produce it being the existence of inflammation or periostitis.

To convince you thoroughly, I am prepared to make the experiment with my method before your honorable Congress.

